# Leveraging Rural Energy Investment for Parasitic Disease Control: Schistosome Ova Inactivation and Energy Co-Benefits of Anaerobic Digesters in Rural China

**DOI:** 10.1371/journal.pone.0004856

**Published:** 2009-03-18

**Authors:** Justin Remais, Lin Chen, Edmund Seto

**Affiliations:** 1 Department of Environmental and Occupational Health, Rollins School of Public Health, Emory University, Atlanta, Georgia, United States of America; 2 Institute of Parasitic Disease, Sichuan Provincial Center for Disease Control and Prevention, Sichuan, People's Republic of China; 3 Center for Occupational and Environmental Health, School of Public Health, University of California, Berkeley, California, United States of America; University of California Los Angeles, United States of America

## Abstract

**Background:**

Cooking and heating remain the most energy intensive activities among the world's poor, and thus improved access to clean energies for these tasks has been highlighted as a key requirement of attaining the major objectives of the UN Millennium Development Goals. A move towards clean energy technologies such as biogas systems (which produce methane from human and animal waste) has the potential to provide immediate benefits for the control of neglected tropical diseases. Here, an assessment of the parasitic disease and energy benefits of biogas systems in Sichuan Province, China, is presented, highlighting how the public health sector can leverage the proliferation of rural energy projects for infectious disease control.

**Methodology/Findings:**

First, the effectiveness of biogas systems at inactivating and removing ova of the human parasite *Schistosoma japonicum* is experimentally evaluated. Second, the impact of biogas infrastructure on energy use and environmental quality as reported by surveyed village populations is assessed, as is the community acceptance of the technology. No viable eggs were recovered in the effluent collected weekly from biogas systems for two months following seeding with infected stool. Less than 1% of ova were recovered viable from a series of nylon bags seeded with ova, a 2-log removal attributable to biochemical inactivation. More than 90% of *Ascaris lumbricoides* ova (used as a proxy for *S. japonicum* ova) counted at the influent of two biogas systems were removed in the systems when adjusted for system residence time, an approximate 1-log removal attributable to sedimentation. Combined, these inactivation/removal processes underscore the promise of biogas infrastructure for reducing parasite contamination resulting from nightsoil use. When interviewed an average of 4 years after construction, villagers attributed large changes in fuel usage to the installation of biogas systems. Household coal usage decreased by 68%, wood by 74%, and crop waste by 6%. With reported energy savings valued at roughly 600 CNY per year, 2–3 years were required to recoup the capital costs of biogas systems. In villages without subsidies, no new biogas systems were implemented.

**Conclusions:**

Sustainable strategies that integrate rural energy needs and sanitation offer tremendous promise for long-term control of parasitic diseases, while simultaneously reducing energy costs and improving quality of life. Government policies can enhance the financial viability of such strategies by introducing fiscal incentives for joint sanitation/sustainable energy projects, along with their associated public outreach and education programs.

## Introduction

Cooking and heating remain the most energy intensive activities among the world's poor, and thus improved access to clean energies for these tasks has been highlighted as a key requirement of attaining the major objectives of the UN Millennium Development Goals [1]. For many, a move towards clean energy technologies for these activities involves a switch from biomass to modern liquid and gaseous fuels. Biogas is an example of a renewable fuel that is well poised to replace biomass fuels in rural settings where organic human and animal wastes are abundant. The present large-scale investment in rural biogas energy in India, Pakistan, Bangladesh, China and elsewhere [2]–[5] has the potential to provide immediate benefits, including the control of neglected tropical diseases (NTD). Here, we argue that by leveraging the proliferation of biogas energy for NTD control, the public health sector has an opportunity to simultaneously achieve multiple development goals that are currently uncoordinated.

Digestion of organic waste material under anaerobic conditions generates “biogas,” the primary constituent of which is methane which can be used for cooking, heating and lighting. China provides a prime example of the rapid investment in this simple technology, as government financial support for rural biogas projects, funded mainly by the Ministry of Agriculture, has increased from 1 billion CNY in 2003 to over 2.5 billion CNY in 2006 [6] (approx. 7 CNY = 1 USD). In 2005, China's National People's Congress passed the Renewable Energy Law, which aimed to remove market barriers and establish a national financial guarantee system for renewable energy projects, with an emphasis on rural energy infrastructure. Biogas construction subsidies are available to households ranging between 800 and 1,200 CNY [7]. Adoption of biogas systems has responded accordingly, increasing from 9.8 million households in 2000 to nearly 18 million in 2005, with the goal of 27 million by 2010. A national target has been set to more than double the supply of biogas fuel in China, from 19×10^9^ m^3^ to 48×10^9^ m^3^ by 2020 [8], [9].

To support the rapid adoption of these systems among households, various benefits of their use have been cited including improved rural sanitation, reduced labor requirements for wood collection (especially among women), reduced greenhouse gas emissions, improved respiratory health in kitchens, and increased agricultural productivity through improvements in soil quality [10]–[13]. While a number of these benefits have been supported by quantitative analyses such as greenhouse gas mitigation calculations [12], [14] and indoor air quality measurements [15], benefits related to the control of NTDs remain unmeasured and largely ignored for lack of basic data such as the degree of pathogen removal during anaerobic digestion. In the context of schistosome parasites for example, experimental data on the effect of anaerobic digestion on schistosome ova in household-scale biogas systems are currently unavailable.

What is more, where the diffusion of biogas technology has been limited, a lack of community acceptance of the technology has been faulted [16]–[18]. Thus there is the need to demonstrate not only the basic sanitation capabilities of these systems, but also the degree to which they are valued by participating households. Here, we investigate the co-benefits of a biogas project in Sichuan Province, PRC, considered the birthplace of biogas implementation [19]. The effectiveness of biogas systems at stemming the NTD *Schistosoma japonicum* by inactivating and removing ova in biogas-treated effluent is experimentally evaluated. Second, we assess community acceptance of recent biogas installations, determining the impact of biogas infrastructure on energy use and environmental quality as perceived by surveyed populations in Sichuan.

### Study Region

**Figure 1:**
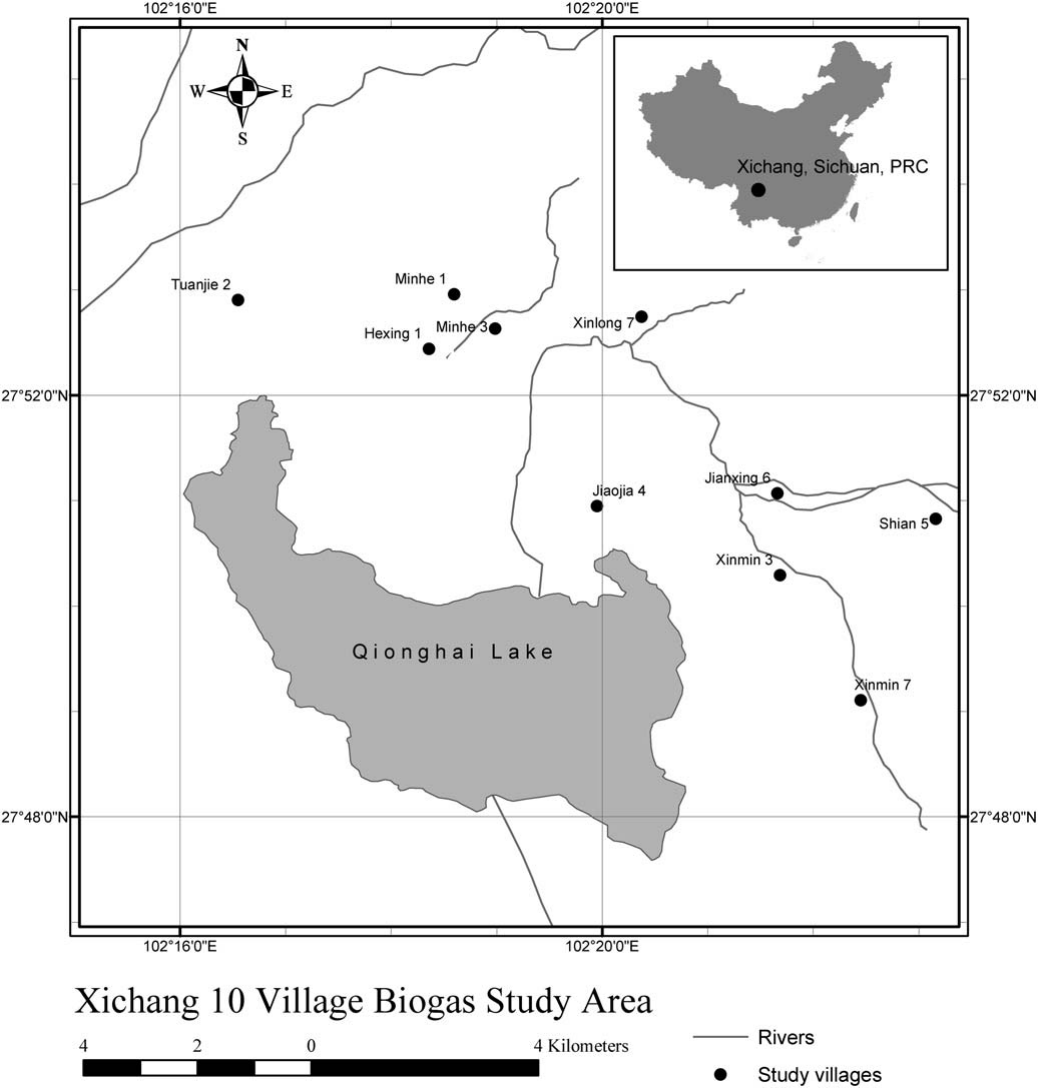
Map of study region showing participating villages near the city of Xichang in Sichuan Province, PRC.

The study was conducted in 10 villages with historically high schistosomiasis infection prevalence in three townships (Daxing, Chuanxing, and Gaojian), near the city of Xichang in Sichuan Province, PRC (E102°18′ N27°52′; Figure 1). The villages lie on the mountainous margins of Qionghai Lake and are characterized by a subtropical climate with an annual average temperature of 18°C and annual rainfall greater than 1,000 mm. Their combined human population is approximately 2,000 individuals, more than 40 percent of whom were infected with schistosomiasis in 2000 [20]. The landscape is dominated by intense, irrigated agricultural cultivation, especially rice, tobacco, wheat and vegetables. Despite most of the villages having access to well-water, sanitation access in these villages remains poor. Contact with potentially schistosome-contaminated water occurs frequently through agricultural work. Moreover, human and domestic animal waste are not typically treated, but stored, and reused as fertilizer. The pervasive use of human waste, termed nightsoil, for crop fertilization in this region leads to the release of parasitic ova into the environment, sustaining schistosomiasis transmission [21]. The dominant factors which govern schistosomiasis transmission in these villages has been studied extensively [20].

Nearly three million household biogas systems are in use in Sichuan, representing nearly a fifth of total biogas systems installed in PRC [22]. The Ministry of Agriculture funds biogas construction in Sichuan through the integrated improvement of kitchens, latrines and livestock sheds. The standard subsidy is 1,000 CNY per family, which is used to purchase cement, equipment and to pay for skilled technicians. Parameters for the design, construction, maintenance and operation of these fixed-dome, 6 m^3^ household systems have been well described elsewhere, backed by more than three decades of research and technical experience from specialized institutions [13], [23]–[36]. The recent biogas program in Xichang County began in 2003, with a total investment of more than 10 million CNY.

## Methods

### Ova Removal Experiments

The main challenge of assessing the effectiveness of biogas systems at removing/inactivating schistosome ova is the extremely low concentration of eggs in the typical system. Owing to this limitation, three separate experimental approaches were undertaken to determine removal in the spring and summer of 2004. These experiments were designed to separately assess removal by biochemical inactivation and removal by sedimentation, both of which are active pathogen removal mechanisms in biogas systems [36]. The first experiment examined the combined effect of these mechanisms in a functioning biogas system. The second experiment examined the isolated effects of biochemical inactivation, and the third investigated removal by sedimentation alone using *Ascaris lumbricoides* eggs as a proxy for *S. japonicum* eggs. All three experiments were conducted in the village Xinlong 7, in systems under conditions of normal use. The experimental details are described below.

In the first experiment, an operating biogas system was seeded with a single pulse of highly infected stool, and the effluent was analyzed weekly for two months (May and June) for the presence of viable ova. The egg density of 2.4 kg of homogenized infected stool was determined to be >100 epg using a triplicate 41.5-mg per slide Kato-Katz thick smear method [37] conducted by an expert microscopist. The stool was input into an operating biogas system, and the effluent was analyzed using a hatch test. This test, which followed the standard Ministry of Health hatch test protocol [38], involved the collection and homogenization of approximately 500 g of effluent from the system, from which three 30 g samples were drawn. Under controlled light and temperature conditions to discourage hatching, samples were sequentially screened to collect eggs using first 40–60 mesh copper, then 260 mesh nylon gauze. Resulting residual material from the nylon screen was placed in a 250 ml Erlenmeyer flask filled with distilled water and incubated. Hatching miracidia, if any, were counted at the meniscus at 6 h, 12 h, and 18 h. Use of the hatch test is preferred here for its ability to detect *viable* ova, which microscopic ova counts cannot provide.

A second experiment conducted in two biogas systems was designed to quantify the biochemical inactivation of schistosome ova in biogas chambers. Egg density of a homogenized infected stool sample was determined (epg = 10) by the Kato-Katz thick smear method, and 40 g samples of stool were seeded into nylon mesh bags (*n* = 16). Bags were input into two biogas systems, exposing the eggs to the digestion environment, while identically prepared control bags were stored suspended in stool. Bags were removed at two-week, one-month and two-month intervals, and the resulting contents analyzed by the hatch test described above. Removal (*R_T_*) was calculated as a decimal reduction, adjusted for control recovery, expressed as log_10_ removal among pooled samples from the same exposure group, *T*:
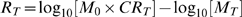
where *M_0_* = miracidia at *t* = 0; *CR_T_*  = control recovery (%) at *t* = *T*; and *M_T_*  = miracidia recovered at sampling time *T*.

A third experiment was designed to isolate removal by sedimentation in biogas systems using *A. lumbricoides* ova present in the digester. While the removal of *A. lumbricoides* eggs is itself an important benefit of biogas systems, here *A. lumbricoides* ova were used as a proxy for schistosome ova because *A. lumbricoides* concentration in stool is about two orders of magnitude higher than schistosome ova [39], and are therefore detectable in both the influent and effluent of typical systems. Ova of *Ascaris* spp. have previously been used as models for helminthes in sewage sludge [40], [41]. To assess the suitability of *A. lumbricoides* ova sedimentation as a proxy for *S. japonicum* sedimentation, the theoretical settling velocities of ova were calculated using known values for ova dimensions and densities, and a modified Stoke's equation following Shuval et al. [42]:
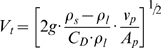
where 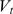 is the terminal velocity(cm sec^−1^); 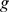 is gravitational acceleration; 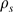 is the ovum density (g cm^−3^); 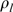 is the liquid density (g cm^−3^); 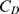 is the drag coefficient (24/Re); 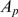 is the ovum projected area (cm^2^); and 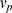 is the ovum volume (cm^3^). Sedimenting ova are thus treated as discrete, nondeformable particles settling in a dilute solution, and it is assumed that terminal velocity is reached instantaneously. Thus, the ratio of terminal settling velocities of *S. japonicum* to *A. lumbricoides* ova was estimated to be 1.34 (indicating the former settles more rapidly than the latter), and therefore the use of *A. lumbricoides* removal as a proxy for *S. japonicum* removal by sedimentation is expected to slightly underestimate removal of *S. japonicum* ova in the systems by this mechanism. Samples (500 g) were drawn from the influent and effluent of two biogas systems and quantitatively screened for *A. lumbricoides* ova using the above-described Kato Katz method every two weeks for three months to estimate the removal of eggs by sedimentation. The hydraulic residence time of each system was calculated using estimates of tank volume and volumetric input and output rates. *A. lumbricoides* removal was calculated as a decimal reduction between samples drawn one residence time apart.

### Study population and surveys

In 2003, 33 heads of household representing a total of 162 household members in two schistosomiasis endemic villages, Xinlong 7 and Xinming 3, were randomly selected to participate in a pre-biogas questionnaire to profile their current energy usage, and to assess, using multiple measures, the perceived value of implementing biogas in their homes. In 2007, a follow-up, post-biogas survey was conducted in 10 endemic villages (which included the 2 villages from the pre-biogas survey) in Xichang County with approximately 25 heads of households randomly selected in each, representing approximately 875 household members. Participants were asked again about their energy usage, whether they had a household biogas system, and if so, how the system was being used and their satisfaction with the system's operation. To examine the influence of the ongoing government program, the larger survey was designed to be more comprehensive, including villages that were and were not included in the biogas subsidy program. All surveys were administered with the free and informed consent of participants by trained personnel from the Sichuan Institute of Parasitic Diseases and the Xichang County Schistosomiasis Control Station.

Socio-demographic data were obtained from a baseline survey of household characteristics conducted in 2000, as part of a larger epidemiologic study of schistosomiasis transmission in these villages. These data were used to explore differences in sociometric variables between villages. No significant differences existed between villages with respect to gender ratio (male∶female of 51∶49), age structure (23%<14 years, 29% 14–29 years, 37% 30–49 years, and 11% 50+ years), education (12% illiteracy, 85% having at least an primary school education), and occupation (the majority, 58.3%, being farmers). Household characteristics such as median numbers of individuals per household (3–4 individuals) and median numbers of pigs per household (2–3 pigs) were consistent between villages, while median household incomes ranged from 1,500 to 7,500 CNY. Logistic regression was used to evaluate the effect of pig ownership, household size, and income on interest in biogas in the pre-biogas survey, and on the presence of biogas in the post-biogas survey.

### Ethical considerations

The study was approved by the Committee for the Protection of Human Subjects at the University of California at Berkeley, and the Institutional Review Board of the Sichuan Centers for Disease Control, Chengdu, PRC, prior to data collection. Written informed consent was obtained from all study participants, and all research procedures were conducted according to the principles expressed in the Declaration of Helsinki.

## Results

### Ova removal experiments

No viable eggs were recovered in the effluent collected weekly from two biogas systems for two months following seeding with highly infected stool. Likewise, zero viable eggs were recovered (no hatched miracidia observed, *M_T_* = 0) from nylon bags removed from two biogas systems after 1, 1.5 or 2 months of exposure. However, adjusted for control recovery, less than 1% of ova were recovered viable (*M_0_* = 1068; *CR_T_* = 77%; *M_T_* = 8) from nylon bags exposed to the biogas chambers for two weeks, a greater than 2-log removal attributable to biochemical inactivation. Furthermore, 91% of *A. lumbricoides* ova counted at the influent of two biogas systems were removed from (not present in) effluent samples when lagged by hydraulic residence time, *T*, (*M_0_* = 7417; *M_T_* = 568), approximately 1-log removal attributable to sedimentation. In both the seeding and the proxy experiments, there were no significant differences between the removal estimates of the two systems. Hydraulic residence times ranged from 14 to 36 days. A combined estimate of 3-log removal accounting for both biochemical inactivation and sedimentation suggests that biogas infrastructure can strongly reduce parasitic ova contamination resulting from nightsoil use.

### Pre-biogas survey

The pre-biogas survey in two villages in 2003 revealed that participants made use of diverse fuels for daily activities such as lighting and cooking (Table 1). Furthermore, while no households surveyed in 2003 had biogas systems, more than a third of participants (37%) knew of someone who has a system, and of those, 88% and 83% rated that system as operating well and a good investment, respectively. Of those surveyed, 69% indicated interest in having a biogas system installed in their household, citing various reasons for their interest, while those lacking interest indicated construction cost as the primary reason (Table 2). No association was found between size of household or household income and interest in, or opinions about, biogas systems.

**Table 1:** Pre-biogas survey of energy usage by type.

	Percentage of households using energy source
Lighting	
Electricity	98
Candles	10
Kerosene	7
LPG	2
Cooking	
Electricity	2
Kerosene	5
LPG	5
Coal	88
Wood	57

**Table 2:** Pre-biogas survey of reasons cited by households for their interest or lack thereof in acquiring a biogas system.

Percentage who cited as their primary reason…	
…for wanting a biogas system	
Inexpensive energy	79
Improved health	14
Improved quality of fertilizer	7
…for not wanting a biogas system	
Expense of construction	70
Expense of upkeep/maintenance	7
Reliability	7
Reduced fertilizer quality	7
Not enough animals	7

Positive interest in biogas was associated with an increased number of pigs in the household. Indeed, gas production is largely determined by the quantity of input waste, pigs being the dominant source by mass [36]. The average number of pigs reported per household was 2.6 (range 0–8). For each increase of one household pig, the probability of positive interest in a biogas system increased by 24% [95% CI 6–163%, p<0.03].

Respondents in the pre-biogas survey estimated the cost of biogas installation at 2,100 CNY (range 800–8,000 CNY). Two-thirds of respondents in the pre-biogas survey indicated that they would be willing to pay 800 CNY (approximately US $100 at the time of survey) for a system, and as would be expected, this group nearly exactly coincided with respondents indicating interest in installing a system in their home. Half of those with interest in a biogas system expressed the need for a loan to help cover the costs of construction.

### Post-biogas survey

A total of 254 households in 10 villages participated in the post-biogas survey. Of these, 54 households (21%) had biogas systems. Biogas systems were found in half of the villages surveyed, and only in villages where government subsidies were available (Table 3). The exception was in Chuanxing Hexing 1 village where two older systems built in the 1970s were reported, but were no longer in use. With the exception of five systems (the two systems in Hexing 1, a system in a household where all pigs had just been slaughtered, and two systems where the households had just finished the installation), all systems produced biogas, and were being used. The systems in use were built between 2002 and 2006.

**Table 3:** Post-biogas survey participants and biogas usage.

Village	Respondents	# have biogas	%
Daxing Xinming 3*	26	18	69
Daxing Xinming 7*	25	6	24
Daxing Shian 5*	25	10	40
Daxing Jianxing 6	25	0	0
Chuanxing Jiaojia 4	26	0	0
Chuanxing Hexing 1	25	2	8
Chuanxing Minhe 1	26	0	0
Chuanxing Minhe 3	26	0	0
Chuanxing Xinlong 7*	25	18	72
Gaojian Tuanjie 2	25	0	0
Total	254	54	21

* Villages where biogas subsidies were made available.

Every household with a biogas system in use reported that they received a subsidy to build their system. These subsidies were valued at 1,000–1,500 CNY, with one household reporting only 300 CNY. On average villagers estimated the total cost of each system to be 2,900 CNY (range 1,300–14,000 CNY), and they estimated that on average they save approximately 600 CNY (range 100–1,000 CNY) per year in reduced household energy costs. Because subsidies were provided in the form of construction supplies and accessories such as pressure gauges and biogas appliances (e.g. rice cooker, single-burner range), the installation of a biogas system was often associated with other household improvements; 98% of the families installed improved toilets at the same time as their biogas system. Since most of the systems were relatively new, no maintenance costs were reported, except for one respondent who spent 40 CNY to fix a system, but ultimately stopped using the system altogether.

The dominant reason provided in the post-biogas survey for why a household did not install a biogas system was not cost, but rather lack of sufficient space on their property to build a system. There were no significant differences in household incomes between those having systems versus not having systems. Similarly, there was no significant difference in average educational level between those with and without biogas.

### Usage and energy benefits

The typical household biogas system relies upon the input of both human and pig waste into the system to produce biogas. On average for those using biogas there were 4.7 persons in the household (range 2–9) and 2 pigs (range 0–5). Some households (38% of those surveyed) reported that they had to purchase additional pigs shortly after the system was installed to generate sufficient quantities of biogas. When we administered our survey, nearly all families reported that their system produced sufficient biogas to fuel their desired use of biogas-operated appliances. The only family not using their system reported a system leak and insufficient gas production.

Nearly all households (98%) reported using biogas fuel for cooking, which is consistent with the subsidy program that provided kitchen appliances at no cost. Prior to using biogas many households used a combination of energy sources for cooking. Households reported using some wood (94%), coal (49%), crop waste (2%), and electricity (28%; sum greater than 100% due to use of multiple fuels). All respondents reported that switching to biogas resulted in cleaner and less smoky kitchens, and that cooking with biogas was easier than using their previous cooking fuel. On average, respondents' systems produced sufficient biogas to support 1.2 hours of cooking time per day (range 0.2–3 hours).

While we are aware of the use of biogas for lighting in other counties in Sichuan Province, in the villages surveyed here, no households reported this use. This may reflect the fact that lighting equipment was not supplied as part of the subsidy program. What is more, the subtropical summer climate and reduction in biogas production in winter months precludes use of biogas fuel for household heating.

In most households, effluent from the biogas digester is used as a natural fertilizer for agriculture. All respondents felt that there was a sufficient quantity of fertilizer produced by their systems. Most households (82%) reported that the quality of the fertilizer was better having passed through the biogas system, 16% felt it to be roughly the same as pit-stored stool, and 2% felt it to be inferior. Villagers attributed large changes in fuel usage (by mass) to the installation of biogas systems, including a 68% decrease in household coal usage, 74% reduction in wood, and 6% drop in crop waste. In contrast, use of electricity was reported to increase by 3% (by CNY) subsequent to biogas installation, possibly a result of the introduction of modern electrical devices and appliances into villagers' homes.

### Perceptions

Overall, 96% of respondents were satisfied with their decision to build the system. The primary and secondary reasons for a household's decision to implement the biogas system are listed in Table 4. Although improved health and sanitation ranked quite highly among villagers' perceptions, when heads of households were directly asked if they were aware of the benefits of their biogas system in reducing parasitic diseases common in these villages, only 63% responded that they were aware of such benefits.

**Table 4:** Post-biogas perceptions of household biogas system.

	Percentage of households
Primary reason for implementing a biogas system	
Inexpensive energy	48
Improved health and sanitation	42
Convenience and saving time in cooking	6
Subsidies	4
Secondary reason for implementing a biogas system	
Improved health and sanitation	43
Inexpensive energy	23
Improved quality of fertilizer	19
Convenience and saving time in cooking	11
Subsidies	4

The post-biogas survey responses were largely consistent with those of the pre-biogas survey in identifying energy costs, health, and subsidizing construction costs as important factors in determining whether to implement a biogas system. The one difference, however, was that the post-biogas survey also identified convenience as an important benefit to biogas.

## Discussion

Private benefits to households in terms of the consumptive use of biogas for cooking have been emphasized and estimated in various regions, including China [43]–[48], yet some have argued that systems justified solely on this basis are undervalued, suggesting the need to account for community benefits [49]. A notable result of the work reported here is the estimated 1–2 log removal of schistosome ova by two mechanisms in biogas systems with typical residence times. A combined 3-log removal by both mechanisms is possible under the reasonable assumption that the two mechanisms act independently on influent ova. What is more, the separate removal estimates are themselves conservative measures.

Data were available to quantify biochemical inactivation at *t* = 14 days (at longer intervals, no viable ova were detected), considerably shorter than typical residence times. Concentrations of viable ova exposed for longer periods were below the limit of detection, suggesting that biogas systems would exhibit greater than 2-log removal for this mechanism under typical operating conditions. Likewise, removal by sedimentation as estimated by *A. lumbricoides* is conservative, as the terminal settling velocity of *S. japonicum* ova exceeds that of *A. lumbricoides*, and thus the removal estimated experimentally is likely an underestimate. The observed 1-log removal by sedimentation agrees with early removal estimates of unspecified “parasite ova” in similar systems in China [50]. The timescale in which dramatic removal by biochemical inactivation occurred in the present study (14 days) agrees with previous estimates that examined the number of summer days ova survived in biogas chambers [51]. What is more, biochemical inactivation of other pathogens in anaerobic digesters is well established, and key mechanisms of inactivation have been identified elsewhere [e.g. 52], [53], including increased temperature, stress induced by the matrix environment (e.g. pH), microbial predation and competition and inhibition by volatile fatty acids.

As is common in experimental removal studies, this study was limited by a small sample of biogas systems in which removal was estimated. In cases where government subsidies are applied, the Ministry of Agriculture plays an active role in overseeing the design and construction of the systems, which may reduce the variability in removal rates among systems by ensuring uniform design and construction quality. While evaluation of a larger number of systems across multiple seasons would increase confidence in the estimates presented here, our findings in a limited number of systems suggest that the development of biogas infrastructure in endemic areas could offer a significant reduction in parasitic ova contamination from nightsoil use, with important implications for human disease. Simulation studies based on the results presented here suggest the community benefit of reduced worm burden is almost linearly proportional to biogas coverage: a 10% reduction in eggs in a village for 1 year, for example, resulted in a 12% infection intensity reduction in comparison with no control measures [54]. In the continued absence of a viable vaccine for schistosomiasis, increasing the biogas capacity for egg removal may offer a novel, sustainable control option for limiting human infections in this region [55]. Clearly, the fraction of pathogens, including schistosomes, which survives anaerobic digestion and are present in biogas effluent remain a concern, and further treatment is desirable.

Although they were developed collaboratively with the local schistosomiasis control authorities, the surveys in this study lack rigorous validation that would make them useful in different regions of China. Moreover, we acknowledge the limitations of a direct comparison of the pre- and post-biogas surveys. The differences in number of villages and minimal overlap in households between the two surveys did not allow for a formal paired comparison of household perceptions. Thus, our results are meant to be descriptive. We note, however, that the reported reductions in household coal, wood, and crop waste use for energy were based solely on the post-biogas questionnaire, which asked the relatively larger sample of households with biogas systems about energy usage the year before and after the installation of the biogas system. Still, economic development may confound comparisons of energy usage before and after the installation of biogas. An indicator of this development was electricity usage, which increased a modest 3%. Moreover, the post-biogas survey, with its larger sample of households allowed us to assess the association between objective household metrics (particularly household income) and adoption of biogas systems.

The most striking finding from the post-biogas survey was the importance of subsidies in implementing the systems at the village level. In villages without the subsidies, no new biogas systems were constructed. Interestingly, the respondents did not perceive the subsidies to be a critical factor in their decision to implement a system. This suggests that, along with the monetary subsidy, the outreach, education and marketing of biogas benefits that occur with an organized governmental program are key to community adoption.

While the subsidies covered a portion of the construction cost, villagers were still required to pay a considerable amount of money to implement these systems. On average, these systems cost roughly 23–32% of a household's estimated 6,000 CNY annual income. With energy savings valued at roughly 600 CNY per year, 2–3 years are required to recoup the capital cost incurred by a household. Villagers indicated their belief that their systems would last a long time (mean response was 14 years). Moreover, a large percentage of respondents (89%) reported their system still operated as well as when they first had it installed.

Biogas systems offer a number of benefits which are highly valued in this community despite the capital requirements of installation. Villagers perceive both improved sanitation and cleaner kitchens (especially with respect to black carbon) associated with biogas system use. Indeed, the health benefits from improvements in indoor air quality resulting from reductions in indoor burning of coal, wood, and crop residues benefits have been shown elsewhere to be considerable [56], [57]. Participant perceptions of improved fertilizer quality from anaerobic digestion are consistent with previous research that has found high levels of bioavailable nitrogen in biogas residues [58].

### Integrating energy and health policies

The focus of schistosomiasis control in China has been on chemotherapy and control of the intermediate snail host [59], both of which have much to contribute. Yet drug- and molluscicide-based strategies are challenging to sustain, and reducing control effort raises the risk of re-emergence in formerly controlled areas [60]. Current investment in rural energy infrastructure in China can be targeted such that the co-benefits of biogas installations can be maximized, including reducing the transmission of schistosomiasis by targeting endemic and re-emerging areas, where appropriate. Sustainable strategies that integrate rural energy needs and sanitation offer tremendous promise for long-term reduction of parasitic diseases in China, while simultaneously reducing energy costs and improving quality of life. Government policies can enhance the financial viability of such strategies by introducing fiscal incentives for joint sanitation/sustainable energy projects, along with their associated outreach and educational programs.
